# Assessing the Evidential Value of Mental Fatigue and Exercise Research

**DOI:** 10.1007/s40279-023-01926-w

**Published:** 2023-09-08

**Authors:** Darías Holgado, Cristian Mesquida, Rafael Román-Caballero

**Affiliations:** 1 Department of Experimental Psychology, and Mind, Brain and Behavior Research Center, University of Granada, Granada, Spain; 2 Institute of Sport Sciences, University of Lausanne, Quartier UNIL-Centre, Bâtiment Synathlon, Lausanne, Switzerland; 3 Centre of Applied Science for Health, Technological University Dublin, Tallaght, Ireland

## Abstract

It has often been reported that mental exertion, presumably leading to mental fatigue, can negatively affect exercise performance; however, recent findings have questioned the strength of the effect. To further complicate this issue, an overlooked problem might be the presence of publication bias in studies using underpowered designs, which is known to inflate false positive report probability and effect size estimates. Altogether, the presence of bias is likely to reduce the evidential value of the published literature on this topic, although it is unknown to what extent. The purpose of the current work was to assess the evidential value of studies published to date on the effect of mental exertion on exercise performance by assessing the presence of publication bias and the observed statistical power achieved by these studies. A traditional meta-analysis revealed a Cohen’s *d*_*z*_ effect size of − 0.54, 95% CI [− 0.68, − 0.40], *p* < .001. However, when we applied methods for estimating and correcting for publication bias (based on funnel plot asymmetry and observed *p*-values), we found that the bias-corrected effect size became negligible with most of publication-bias methods and decreased to − 0.36 in the more optimistic of all the scenarios. A robust Bayesian meta-analysis found strong evidence in favor of publication bias, *BF*_pb_ > 1000, and inconclusive evidence in favor of the effect, adjusted *d*_*z*_ = 0.01, 95% CrI [− 0.46, 0.37], *BF*_10_ = 0.90. Furthermore, the median observed statistical power assuming the unadjusted meta-analytic effect size (i.e., − 0.54) as the true effect size was 39% (min = 19%, max = 96%), indicating that, on average, these studies only had a 39% chance of observing a significant result if the true effect was Cohen’s *d*_*z*_ = − 0.54. If the more optimistic adjusted effect size (− 0.36) was assumed as the true effect, the median statistical power was just 20%. We conclude that the current literature is a useful case study for illustrating the dangers of conducting underpowered studies to detect the effect size of interest.

## Key Points

**Table:** 

For most of the publication bias methods, there was evidence in favor of selective reporting and inconclusive evidence in favor of the effect, and the effect is substantially reduced after the correction.
Assuming the meta-analytic effect size as the true effect size, most studies had underpowered designs to detect it.
The presence of publication bias and studies with underpowered designs render the effect of mental exertion on exercise performance inconclusive.

## Introduction

Mental fatigue has attracted the attention of many sport scientists over the course of the last two decades given the potential negative consequences for exercise performance [1–3]. A key hypothesis in this area is that performing a cognitive task with high demands increases the subjective feeling of mental fatigue, hindering performance in a subsequent physical exercise task [4]. This hypothesis relies mainly on two assumptions: (1) cognitive tasks with high demands induce a state of mental fatigue and (2) that state of mental fatigue alters subsequent perception of effort during the physical exercise, thereby reducing the amount of effort individuals are willing to expend [4, 5]. While a cursory look at the literature seems to provide strong support for the mental fatigue hypothesis [2, 5], these conclusions may be nuanced by theoretical and methodological caveats. We first delve into the theoretical issues, and then focus on the evidential value of the empirical literature, which is the main aim of this article.

In the context of this article,^1^ mental fatigue can be defined as the feeling of being unable to continue performing optimally, likely due to depletion of the needed resources to accomplish the goals of the cognitive task. This may be accompanied by the feeling of the need to rest, to abandon the demanding task, or at least to switch to an easier one [6]. This may or may not translate into an objective decrease in cognitive performance, as compensatory mechanisms could be put in place to keep fulfilling the task goals [9]. In fact, subjective and objective indexes of mental fatigue do not always correlate [10, 11]. Furthermore, the emergence and degree of mental fatigue might depend on several factors [12] such as the goal, motivation, expectations and executive capacity of the individual, the (objective and perceived) difficulty of the task, and its duration [13].

The complex relationship between cognitive processing and mental fatigue seems seldom considered in the majority of empirical studies looking at the effect of mental exertion^2^ on subsequent physical performance. First, they assume that performance in a cognitive task induces mental fatigue, and confirm that by reporting participants’ experience of mental fatigue by means of subjective scales (e.g., visual analog scale). However, individuals may not be able to accurately assess their cognitive states because of their limited metacognition, social desirability biases, and their variability when mapping sensations to ratings [12]. This may hence limit the interpretation of the subjective outcome recorded after cognitive tasks in these studies [14]. Second, the employment of standardized cognitive tasks (e.g., Stroop task) should be considered as a limitation because of their lack of individualization and level of engagement [12, 15]. For example, if the task is too difficult for them, the individual may become overwhelmed or frustrated, but not mentally fatigued [16]. Conversely, if the task is too easy, the individual may become bored and lose interest [16]. Furthermore, in the control conditions, tasks with theoretically lower cognitive demands or documentaries are often included, but they differ not only in terms of mental demands compared with the experimental task but also in terms of boredom and engagement [14, 17]. All this can have an impact on subsequent physical performance, but would not necessarily be accompanied by, or linked to, mental fatigue. Related to that, the eventual reduced overall cognitive performance in the experimental condition with respect to the control condition (when two difficulty levels are used), or a more pronounced decline in performance over time, might not be indicative of heightened mental fatigue. Finally, even if the tasks are prolonged over time, the decrease in resources or increase in mental fatigue might not necessarily be associated with different exercise performance effects [1]. This could be explained by the fact that performing a cognitive task is a dynamic experience, albeit this has not yet been tested empirically [18]. For example, it may become easier and require less effort as the task progresses, to eventually be automated, although still requiring effort to stay focused despite boredom. In other words, performance-related factors such as motivation and effort might be more important than the duration of the task. All in all, these shortcomings challenge, or at least nuance, the interpretation regarding the effects of mental exertion on exercise performance in these studies based on the idea of mental fatigue.

Research on mental exertion and physical performance has some similarities with research conducted in the domain of ego depletion [19]. Ego depletion is based on the idea that all acts of willpower and self-regulation deplete a limited pool of resources, impairing performance in a subsequent (cognitive) task. Although this theory has been the cornerstone of more than two decades of research on self-control, the existence of the effect has been questioned in recent years (for a review, see Vadillo, 2020 [20]). For instance, a multilab replication project found that the size of the ego-depletion effect was small with 95% confidence intervals encompassing zero (*d* = 0.04, 95% CI [− 0.07, 0.15]) [21], and other research has pointed to the presence of strong publication bias in the literature [22].

Equally, there have been recent accounts challenging the strength of the mental exertion–exercise performance effect [3, 23]. For instance, the only preregistered study that has attempted to replicate the seminal study by Marcora et al. [24], failed to replicate these findings [23]. After watching a 90 min documentary or performing a mental exertion task (AX-CPT), 30 participants (in comparison with the 16 participants of the original study) completed a time-to-exhaustion cycling task. There was no evidence of reduced performance or increased perceived effort during the cycling task in the mental exertion condition [23]. Nonetheless, the fact that an original finding cannot be replicated does not mean that it does not exist, since science relies on the accumulation of evidence [25].

In some cases, replications do not succeed because of inadequate replication methods or because of factors that moderate the results. However, original studies might not be replicated for a few other reasons. First, because there is no effect to be found [21]. Second, the use of questionable research practices such as *p*-hacking or optional stopping can overestimate the true effect size and lead to a large number of type 1 errors in the published literature after selecting for statistical significance [26–28]. Third, the presence of publication bias in combination with studies using underpowered designs can also distort the cumulative evidence [27, 29, 30]. For example, Wolff et al. [31] conducted a survey where, on average, 16% of respondents (277 out of 1721) had published over three studies on ego depletion, and had completed more than two additional, unpublished studies. On the other hand, if studies are conducted with underpowered designs, the sampling error can cause large swings in effect size estimates [29, 32]. Indeed, studies with underpowered designs will only reach statistical significance if the study happens to yield an overestimated effect size. Indeed, most of the studies in the literature of mental exertion-exercise are based on low sample sizes (mean = 15, *SD* = 9.14; min = 8, max = 63) suggesting that some studies might have underpowered designs to detect a range of hypothetical small and medium effect sizes. For instance, assuming a true Cohen’s *d* effect size of − 0.49 and a within-subject design, a study would require a sample size of 35 participants to achieve a statistical power of 80%.

The presence of these biases is problematic for the credibility of research because it reduces the evidential value of published literature, leading to overestimated meta-analytical effect sizes [33, 34]. For instance, the results of a systematic review and meta-analysis [3] on the effect of mental fatigue on exercise performance seemed to indicate a significant negative effect (*d*_*z*_ = − 0.48, 95% CI [− 0.70, − 0.28]), but a bias-sensitive analysis suggested that after adjusting for publication bias, this estimate was significantly smaller (*d*_*z*_ = − 0.14, 95% CI [− 0.46, 0.16]) [3]. The evidential value of a literature body is determined by the number of studies examining true and false effects, the power of the studies that examine true effects, the frequency of type I error rates (and how they are inflated by *p*-hacking), and publication bias [35, 36]. Given that the likely presence of studies with underpowered designs has been overlooked, there is therefore uncertainty as to whether the published literature on this topic has provided reliable estimates of the effect of mental exertion on exercise performance, whatever the cause of the effect.

One way to assess the evidential value of a body of literature is by considering the presence of publication bias and studies with underpowered designs. However, meta-analytic effect sizes are often taken at face value without considering the evidential value of the primary studies. Therefore, we considered it pertinent to perform further analysis to examine the evidential value of the studies investigating this topic, as has been done in other sport science areas [37]. Furthermore, to date, meta-analyses on the effect of mental exertion have relied on one [1, 38] or two methods [2, 3] to assess for publication bias and small-study effects. For instance, Giboin and Wolf [1] and McMorris et al. [38] only used Egger’s test and Begg’s test, respectively. Likewise, besides Egger’s test, Brown et al. [2] relied on the fail-safe method (which is known to be outdated and should be avoided) and Holgado et al. [3] used a three-parameter selection model. However, simulation studies investigating the accuracy of publication bias tests have shown that factors such as high heterogeneity and studies with small sample sizes can inflate type I error rates, decrease statistical power, and overestimate or underestimate the meta-analytic effect size to a higher or smaller degree [39–41]. Indeed, Carter et al. [39] argued that no single meta-analytic method consistently outperformed all the others due to the different assumptions underlying these methods. As a result, researchers have been recommended to rely on several publication bias tests [39, 40]. Methods such as robust Bayesian meta-analysis (RoBMA [42]) allow incorporating several approaches into the same analysis without needing to choose among them. In the present manuscript, we therefore examined the evidential value of primary studies included in previous meta-analyses and recent articles that have been published afterwards by assessing the presence of publication bias using several tests and the observed statistical power for a range of hypothetical effect sizes achieved by these studies. We hypothesized that (a) there would be evidence of publication bias and (b) most of the published articles would not have adequate power to detect the estimated meta-analytic effect size.

## Methods

The hypothesis, methodology, and analysis plan for this study were preregistered in the Open Science Framework along with the datasets generated and R scripts required to reproduce both the statistical analyses and figures included in this meta-analysis (https://osf.io/5zbyu/).

### Literature Search

We included studies with within-participants designs from previous meta-analyses investigating the effect of performing a mental exertion task before a physical exercise that provided enough information and fulfilled the inclusion criteria [1–3]. Additionally, given that the last available meta-analysis was published in 2020 [3], studies published afterward and up to May 2022 were also considered. Thus, we conducted a literature search for new studies through Medline, Scopus, and Web of Science in May 2022. We used four search terms related to mental fatigue and another four terms related to exercise: “mental fatigue” OR “cognitive fatigue” OR “mental exertion” OR “ego-depletion” AND “physical performance” OR “exercise” OR “muscle fatigue” OR “sport”.

### Study Selection

Studies were selected on the basis of the following inclusion criteria: (1) available in English, (2) within-participant design, (3) participants completed a mental exertion task of any duration prior to a physical exercise, (4) the main outcome was a measure of exercise performance (e.g., time, distance completed, average power/speed, or total work done), (5) the study provides necessary descriptive information of the main performance outcome. Studies investigating the effect of mental exertion on psychomotor or tactical skills were not included. The list of studies reviewed and the reason for exclusion is available at https://osf.io/5zbyu/.

### Data Extraction

A table containing data extracted from each study can be found at https://osf.io/5zbyu/. The major two major pieces of information for the current study were the study’s effect size and its associated *p*-value. If participants completed more than two experimental conditions, we only considered the control condition and experimental condition (i.e., mental exertion) without other factors (e.g., mental exertion in hypoxia). For each study, the following information was extracted: (1) study design, (2) type of experimental conditions, (3) exercise protocol and type of test, (4) statistical test and level of significance, (5) descriptive statistics (study sample size and mean ± *SD*) for both the experimental and control condition, and (6) the result of the statistical test (e.g., *t*(11) = 7.2, *p* < .001). We contacted authors to request unpublished data under two circumstances. First, when no sufficient statistical information was reported to recompute either the study effect size (i.e., *t*-statistic and sample size) or the *p*-value (i.e., degrees of freedom and *F*-ratio or *t*-statistic). Second, when a study used a factorial design with more than two experimental groups but no pairwise comparison of mental exertion condition and control condition was reported. Only one *t*-value and *p*-value per independent study/sample of participants for the main outcome was extracted to meet the independence criteria. The extracted *p*-value corresponded to the same statistical contrast as the effect size estimate.

### Effect Size Calculation

Because we only included within-participant designs (i.e., the most common design of this literature and also because it allows us to control for individual cognitive differences), we decided to use Cohen’s *d*_*z*_ as our type of effect size estimate. The advantage of doing so is that *d*_*z*_ scores are computed on the basis of the same information that is used to test for statistical significance in these studies (i.e., a paired-sample *t*-test) and, consequently, the confidence intervals of the effect size are more consistent with the *p*-values reported in the original papers. Second, the computation of *d*_*z*_ does not require the correlation between dependent measures since correlation parameters are seldom reported as part of statistical analysis. Thus, all study effect sizes were calculated as Cohen’s *d*_*z*_, representing the standardized mean difference between mental exertion and the control group. Cohen’s *d*_*z*_ was calculated directly from the *t*-value and the number of participants using the formula provided by Rosenthal [43], as follows: *d*_*z*_ = *t*/√n. If a study performed a one-way repeated measures ANOVA for the effect of condition, the *F*-ratio was converted into a *t*-statistic as *t* = √*F*. Equally, if the *t*-value was not available, but the exact *p*-value and sample were, we calculated the *t*-value with the following formula in R: qt(1 − (*p*-value/2), *N*). In addition, we estimated repeated-measures correlations from *t*-values and *F*-values from one-way repeated measures ANOVA to reach an overall repeated-measures correlation that could be imputed in studies with more complex designs (i.e., two-way repeated measures ANOVAs; 14 out of 46). Overall, we could extract the correlations in 28 out of 46 studies and, subsequently, we obtained a meta-analytic Pearson’s *r* of .96, 95% CI .93, .99. By assuming it in studies with more complex designs, we estimated *d*_*z*_ from means and standard deviations.

### *p*-value Recalculation

In the case that the corresponding *p*-value was reported relatively (i.e., *p* < .05), the *p*-value was recomputed for *z*-curve analysis when degrees of freedom and *t*-statistic were reported. In the case where the *t*-test was reported but not the degrees of freedom, degrees of freedom were inferred from the study sample size (*N* − 1). *p*-values were recomputed in Microsoft Excel for Mac version 16.45 using the functions *T.DIST.2T* or *F.DIST.RT* for *t*-tests and *F*-tests, respectively.

### Statistical Analysis

#### Meta-analysis

The meta-analysis was performed using the *metafor* R package [44] in R version 3.6.1 (R Core Team, 2019) and relied on a random-effects model to fit the overall effect size to estimate the average reported effect of mental exertion and to assess heterogeneity in effect sizes. The overall effect size is reported along with 95% confidence and prediction intervals. Heterogeneity across studies was assessed by means of Cochran’s *Q* to test whether the true effect size differs between the studies, Thompson’s *I*^2^ to assess the proportion of total variability due to between-study heterogeneity, and tau-squared (*τ*^2^) as estimate of the variance of the underlying distribution of true effect sizes.

#### Testing for Small-Study Effects and Publication Bias

Because previous research has shown that there is no single publication bias and small-study effects method that outperforms all the other methods under each and every assumption tested [39, 40, 45–47], we used a triangulation approach, also known as sensitivity analysis, where we do not rely on only one single publication bias method, but use multiple publication bias methods instead [39, 48–50]. To test for publication bias, we relied on two types of methods based either on funnel plot asymmetry or reported *p*-values and selection models. Methods based on funnel plot asymmetry were Egger’s regression test, which measures a general relationship between the observed effect sizes and its precision, the skewness test [51], which adds to the Egger’s test (its equivalent *T*_1_) a measure of asymmetry based on the shape of the effect sizes’ distribution (*T*_s_, leading to an independent *p*-value or a common *T*_1_*–T*_s_* p*-value), and the limit meta-analysis (LMA; R package metsense [52]). Among *p-*value methods, we used a Three-parameter selection model with a one-tailed *p*-value cutpoint of .025 (3PSM [53]) and *z*-curve (R package *z*-curve 2.0 [54]). The *z*-curve method allows testing for publication bias by considering whether the point estimate of the observed discovery rate lies within the 95% confidence interval (CI) of the expected discovery rate. If the observed discovery rate estimate lies outside the 95% CI of the expected discovery rate is considered evidence of publication bias [54].

Most of these methods also allow adjusting the observed effect size accounting for publication bias, excepting the skewness test and *z*-curve. Among them, the precision-effect test–precision-effect estimate with standard error (PET–PEESE [50]) represents a conditional procedure to correct the final effect size based on the significance of the intercept in the Egger’s meta-regressive model. For a detailed description of the limitations and assumptions of the methods implemented in the present meta-analysis, readers are referred to Carter et al. [39], McShane et al. [40], Stanley [45], Bartos and Schimmack [54], and Sladevoka [41].

We conducted Egger’s test, PET–PEESE, skewness test, and LMA with Fisher’s *z* for being a variance-stabilizing transformation for the effect size and preventing the artifactual dependence between Cohen’s *d* and its precision estimate [55]. For the Fisher’s *z* transformation, we converted Cohen’s *d*_*z*_ into Cohen’s *d*_rm_ [56] for the sake of equivalency with a two-group standardized mean difference, and Cohen’s *d*_rm_ into Fisher’s *z* [57].

We report three deviations from the preregistered analysis. First, the limit-meta was not included in the preregistered protocol [52]. LMA is based on the concept of increasing the precision of the meta-analytic effect size using a random-effects model that accounts for small-study effects [41]. Second, both *p*-curve and *p*-uniform methods were discarded following recommendations of Carter et al. [39]. These methods result in the overestimation of the true effect size under moderate-to-large heterogeneity. Finally, we conducted a robust Bayesian meta-analysis (RoBMA) [50] as it allows weighting multiple models of publication bias regarding their fit to the evidence, without needing to choose among them. RoBMA yields one single model-averaged estimate of the effect size after simultaneously applying (1) selection models that estimate relative publication probabilities (i.e., selection model) and (2) models of the relationship between effect sizes and their standard errors (i.e., PET–PEESE). RoBMA makes multimodel inferences, which are guided mostly by those models that predict the observed data best, about the presence or absence of an effect. On the basis of previous literature that reflect a prior belief of a substantial effect of mental exertion [1–3], we selected a normal distribution centered at − 0.46 (i.e., mean of the outcomes of the three cited meta-analysis) and with one standard deviation as the prior of the effect in the alternative hypothesis. For the effect belonging to the null hypothesis, we assumed a normal distribution centered at 0 an equal standard deviation.

#### Statistical Power

Several statistical power estimates were calculated using two different methods based on *p*-values and effect sizes. First, we conducted a *z*-curve analysis, which is based on the concept that the average power of a set of studies can be derived from the distribution of *p*-values (see [54] for technical details). This method converts significant and nonsignificant *p*-values reported in a literature into two-tailed *z*-scores, and uses the distribution of *z*-scores to calculate two estimates of average power using finite mixture modeling: the expected discovery rate, which is the percentage of studies predicted to be significant based on the average power of published studies and the expected replication rate, which is the average power of the studies entered, which is also an estimate of the percent of the studies that one would expect to replicate if one performed the studies in exactly the same way as they were done before. Second, we used a range of hypothetical effect sizes to estimate statistical power using the R package metameta [58]. This package allows researchers to estimate the statistical power of the studies included in the meta-analysis by using (a) a range of hypothetical effect sizes and (b) the meta-analytic effect size estimate as the true effect size.

## Results

A total of 50 effect sizes were selected for eligibility, but 4 were discarded because descriptive data were not reported and authors did not provide raw data upon request.^3^ A total of 46 effect sizes from independent samples were included in the meta-analysis [23, 24, 61–98]. A disclosure table containing the list of 50 effects selected for eligibility, including those included in meta-analysis and the literature search output, can be found at https://osf.io/5zbyu/.

### Overall Meta-analysis

**Fig. 1:**
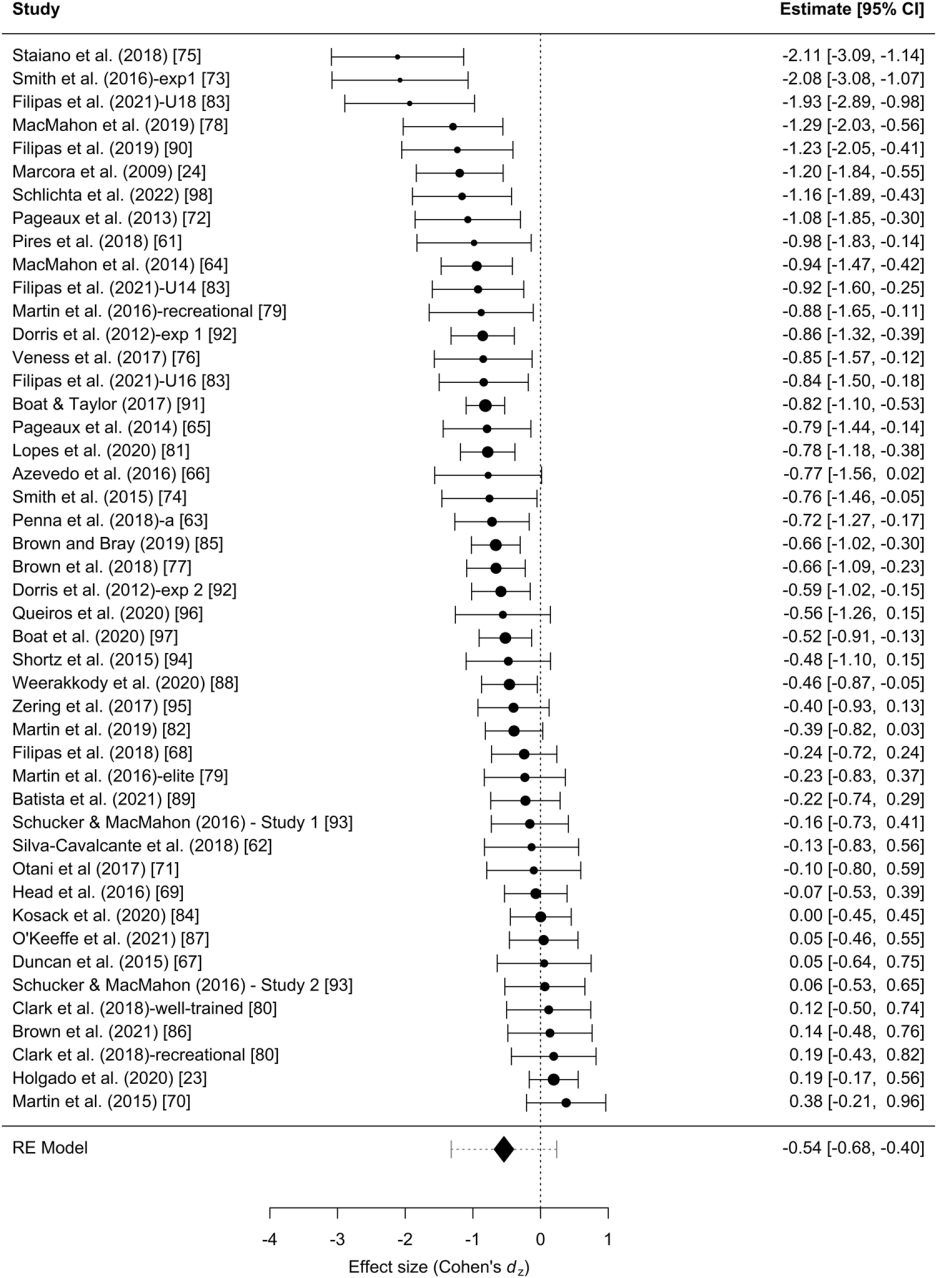
Forest plot of Cohen’s *d*_*z*_ from each individual study and the meta-analyzed effect size [including its 95% confidence interval (width of the rhombus) and its 95% prediction interval (whiskers)]

The results of the random-effects meta-analysis are summarized in Fig. 1. Across all studies, the random-effects meta-analysis revealed a statistically significant meta-analytic effect size of *d*_*z*_ = − 0.54, 95% CI [− 0.68, − 0.40], *p* < .0001. The 95% prediction interval for the meta-analytic effect size was [− 1.32, 0.24]. The significant *Q*-statistic, *Q*(45) = 127.73, *p* < .0001, led us to reject the null hypothesis that all studies share a common effect size. Instead, there was true heterogeneity between studies, suggesting the true effect sizes differed between the studies. The estimated heterogeneity was *τ*^2^ = 0.15. Furthermore, *I*^2^ was 66.58%, indicating that about two-thirds of total variability is due to between-study heterogeneity.

### Small-Study Effects and Publication Bias

**Fig. 2:**
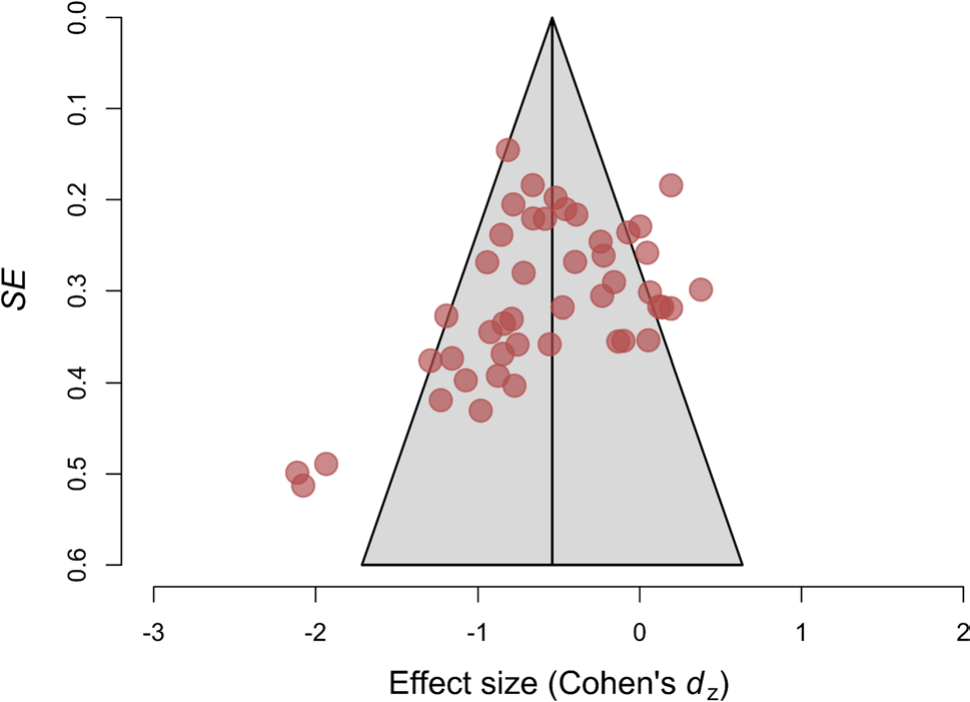
Funnel plot of study Cohen’s *d*_*z*_ effect size versus the study’s standard error

**Fig. 3:**
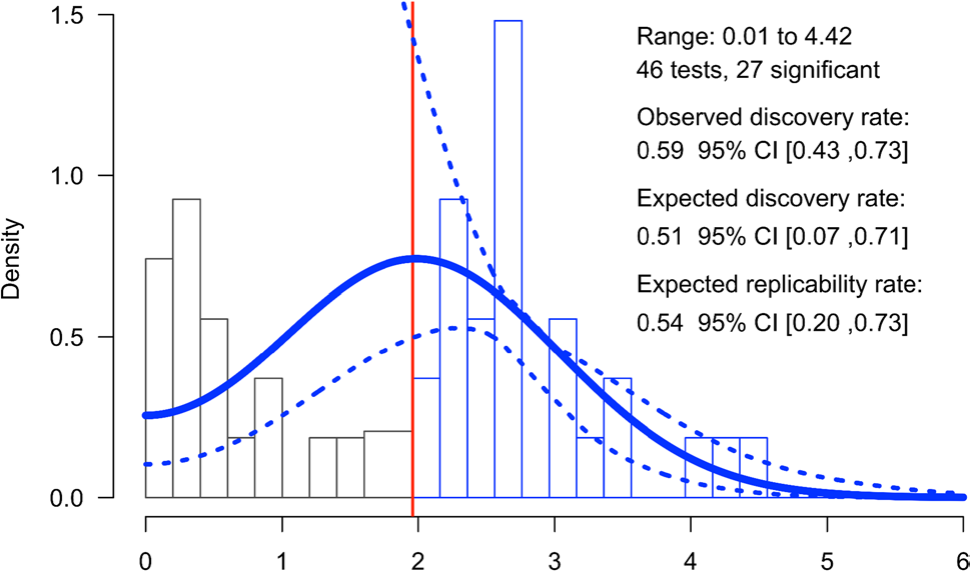
Distribution of *z*-scores. The vertical red line refers to a *z*-score of 1.96, the critical value for statistical significance when using a two-tailed alpha of .05. The dark-blue line is the density distribution for the inputted *p*-values (represented in the histogram as *z*-scores). The dotted lines represent the 95% CI for the density distribution. Range represents the minimum and maximum values of *z*-scores used to fit the *z*-curve. A total of 46 independent *p*-values (27 significant) were converted into *z*-scores to fit the *z*-curve model

Results of small-study and publication bias tests are summarized in Table 1. Although a visual inspection of the funnel plot suggested the existence of asymmetry, the Egger’s regression test using Fisher’s *z* and its standard error yielded a nonsignificant outcome, *b*_1_ = − 0.32, SE = 0.53, *z* = − 0.61, *p* = .542 (Fig. 2). In the same line, the test of small-study effects in LMA was not significant, *Q*(1) = 0.22, *p* = .636. In contrast, the skewness test was significant, *T*_s_ = − 1.00, 95% CI [− 1.77, − 0.23], *p* = .005, along with the combined test, *T*_1_*–T*_s_* p*-value = .011, both indicating asymmetry in the distribution of effect sizes. Similarly, the 3PSM approached a better fit assuming the presence of publication bias based on the study’s *p*-values, although it did not reach the significance level: *χ*^2^(1) = 3.80, *p* = .051. In the *z*-curve (Fig. 3), the observed discovery rate estimate (.59) was numerically larger than the expected discovery rate (.51). However, the observed discovery rate lay within the 95% CI [.07, .71] of the expected discovery rate, suggesting that the available evidence was not sufficient to reject the hypothesis of the existence of publication bias. Finally, RoBMA found strong evidence in favor of heterogeneity, *BF*_rf_ = 659,278.66, and strong evidence in favor of publication bias, *BF*_pb_ = 8403.64. Therefore, most of the methods indicated the presence of publication bias in the reviewed literature, and those that did not reach the significance level offered numerical and visual trends in line with the presence of selective reporting.

**Table 1:** Summary results of publication bias and small-study effects tests

Test	Result	Interpretation
Egger’s test (Fisher’s *z*)	*b*_1_ = − 0.32, *SE* = 0.53, *z* = − 0.61, *p* = .542	Nonsignificant evidence for small-study effects
LMA	*Q*(1) = 0.22, *p* = .636	Nonsignificant evidence for small-study effects
Skewness	*T*_s_ = − 1.00, 95% CI [− 1.77, − 0.23], *p* = .005 *T*_1_*–T*_s_* p*-value = .011	Potential publication bias
3PSM	*χ*^2^(1) = 3.80, *p* = .051	Nonsignificant evidence for small-study effects
*z*-curve	ODR estimate (.59) ∈ 95% CI [.07, .71] of the EDR	Nonsignificant evidence for publication bias
RoBMA	*BF*_pb_ = 8403.64	Potential publication bias

As a subsequent step, we used most of the previous methods to adjust the final effect in the absence of bias. All of the applied methods converged to a reduction of the final effect size, most of them indicating a null outcome (Table 2). PET–PEESE is a two-step procedure whereby only if the null hypothesis is rejected, is the second step PEESE performed to calculate the adjusted meta-analytic effect size [50]. The PET estimate was selected because the procedure returned a nonsignificant effect, adjusted Fisher’s* z* = − 0.06, 95% CI [− 0.25, 0.13], *p* = .561, and, therefore, the null hypothesis of zero effect could not be rejected (versus unadjusted Fisher’s *z* = − 0.11, 95% CI [− 0.17, − 0.07]). Likewise, LMA returned a nonsignificant adjusted Fisher’s* z* = − 0.06, 95% CI [− 0.31, 0.20], *p* = .664 (versus unadjusted Fisher’s *z* = − 0.12, 95% CI [− 0.19, − 0.04]). The fit of the 3PSM returned a significant but substantially reduced adjusted effect size, adjusted *d*_*z*_ = − 0.36, 95% CI [− 0.58, − 0.15], *p* = .001 (versus unadjusted *d*_*z*_ = − 0.54). Finally, RoBMA led to a mostly smaller corrected effect size and inconclusive evidence in favor of the effect, adjusted *d*_*z*_ = − 0.02, 95% CrI [− 0.47, 0.33], *BF*_10_ = 0.90 (from the *d*_rm_ outcome of the model, and using a common *r* = .96).

**Table 2:** Summary results of adjusted effect sizes after publication-bias corrections

Test	Result	Interpretation
PET–PEESE (Fisher’s *z*)	Adjusted Fisher’s* z* = − 0.06, 95% CI [− 0.25, 0.13], *p* = .561 (versus unadjusted Fisher’s *z* = − 0.11, 95% CI [− 0.17, − 0.07])	Null-adjusted effect
LMA	Adjusted Fisher’s* z* = − 0.06, 95% CI [− 0.31, 0.20], *p* = .664 (versus unadjusted Fisher’s *z* = − 0.12, 95% CI [− 0.19, − 0.04])	Null-adjusted effect
3PSM	Adjusted *d*_*z*_ = − 0.36, 95% CI [− 0.58, − 0.15], *p* = .001	Reduced but significant adjusted effect
RoBMA	Adjusted *d*_*z*_ = − 0.02, 95% CrI [− 0.47, 0.33], *BF*_10_ = 0.90 (from the *d*_rm_ outcome of the model, and using a common *r* = .96)	Null-adjusted effect

### Statistical Power

**Fig. 4:**
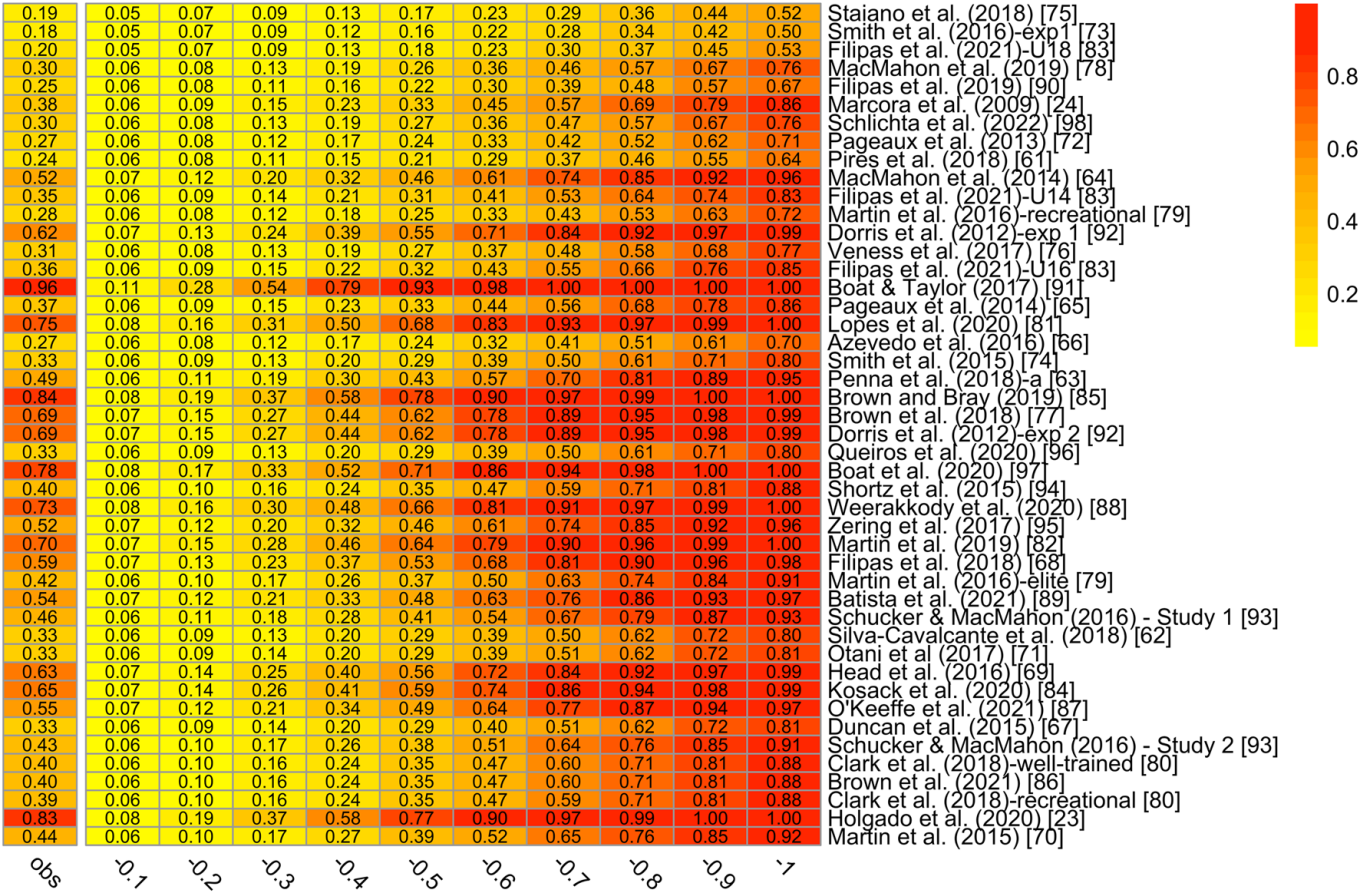
Observed statistical power estimates for studies included in the meta-analysis assuming a range of hypothetical effect sizes [− 0.1, − 1]. The leftmost column (*obs*) refers to the observed statistical power assuming the meta-analytic effect size (*d*_*z*_ = − 0.54). The rest of the columns represent the observed statistical power of each study given a hypothetical effect size

The metameta package [99] was used to calculate statistical power estimates for a range of hypothetical effect sizes. The median statistical power of the studies included in the meta-analysis, using the meta-analytic effect size estimate as the true effect size (*d*_*z*_ = − 0.54) was 39% (min = 19%, max = 96%). The statistical power estimates of the studies included in the meta-analysis for a range of hypothetical effect sizes is shown in Fig. 3. If we assume the only one significant bias-corrected effect size (i.e., 3PSM, *d*_*z*_ = − 0.36) as a true effect size, the median observed statistical power of the studies would be 20% (min = 11%, max = 70%). The *z*-curve method was also used to estimate average statistical power of all studies included in the meta-analysis (see Fig. 4). The expected discovery rate was .51, 95% CI [.07, .71], indicating that the average power of all studies was approximately 51%. The expected replication rate was .54, 95% CI [.20, .73], indicating that the average power of only those studies reporting a statistically significant effect was 54%.

## Key Findings

In the present manuscript, we attempted to examine the evidential value of studies investigating the effect of a mental exertion on a subsequent physical exercise. We hypothesized that (a) there would be evidence of publication bias and (b) most of the published articles would not have adequate power to detect the estimated meta-analytic effect size. Although some tests for funnel plot asymmetry failed to reach a significant result for a general relationship between the observed effect sizes and its precision estimate, such as Egger’s regression test and LMA, the skewness test, which performs well under substantial heterogeneity (i.e., *τ*^2^ = 0.15 and *I*^2^ = 67%), showed evidence of asymmetry based on the shape of the effect sizes’ distribution. The skewness test also contributed with a combination of both measures of asymmetry that suggested the presence of small-study effects in the literature (i.e., *T*_1_*–T*_s_* p*-value = 0.011). Among tests of publication bias based on *p*-values, 3PSM showed a better fit assuming the presence of publication bias, while the *z*-curve could not reject the null hypothesis of no publication bias. Finally, RoBMA, which offers a model-averaged estimate of the effect size after fitting models of both types of approaches (i.e., funnel plot asymmetry and selective reporting based on *p*-values) found strong evidence in favor of publication bias.

Therefore, it seems that there was substantial evidence in favor of selective reporting in the literature of mental exertion on exercise performance. Under those conditions, all methods converged to a substantial reduction of the final effect size, in most cases leading to a null outcome, such as PET–PEESE (adjusted Fisher’s* z* = − 0.06, *p* = .561), LMA (adjusted Fisher’s* z* = − 0.06, *p* = .664), and RoBMA (adjusted *d*_*z*_ = − 0.02, *BF*_10_ = 0.90). RoBMA also found inconclusive evidence in favor of the effect (*BF*_10_ = 0.90). The only method that reported a still significant effect was 3PSM, although it offered a meta-analytic effect size after adjusting for publication bias reduced by at least 0.18 standard deviations (*d*_*z*_ = − 0.36, *p* = .001). On the basis of the results from all the applied methods, it seems reasonable to conclude that the negative effect of mental exertion on exercise performance, if it exists, is likely to be much smaller than reported.

Furthermore, the median observed statistical power assuming the meta-analytic effect size (*d*_*z*_ = − 0.54) as the true effect size was 39% and if the more optimistic adjusted effect size, the one by 3PSM was assumed as the true effect (− 0.36), the median statistical power was 20%. These results are also in line with the results obtained from the *z*-curve analysis which yielded an observed statistical power of 51%, 95% CI [.07, .71], for both significant and nonsignificant results. Both the shrinkage of the meta-analytic effect size estimate after adjusting for publication bias and the presence of underpowered designs might therefore suggest that the evidential value of the studies included in this meta-analysis is low on average.

### The Negative Expectancies About Mental Fatigue Should be Lower

In this study, we analyzed the evidential value of the empirical literature on the effect of mental exertion during a cognitive task has a negative consequence on subsequent physical exercise [24, 63, 65], independent of the causal mechanism responsible for the effect. The meta-analysis results (*d*_*z*_ = − 0.54, 95% CI [− 0.68, − 0.40]) revealed that mental exertion might hinder exercise performance. However, when the overall estimate is adjusted for publication bias, most of the tests provided nonsignificant and a largely reduced adjusted estimate of the true effect of mental exertion on exercise performance, suggesting that the effect might be substantially smaller (see Table 2). For instance, the new meta-analytic effect size was reduced by at least 0.18 standard deviations on the basis of the results from the 3PSM (*d*_*z*_ = − 0.36, *p* = .001). The reasons for publication bias are multiple [100, 101], but it varies from editorial predilection for publishing positive findings, researchers’ degree of freedom in analyzing the data [102], authors not writing up null results, and other causes. The presence of publication bias in a set of published studies is likely to inflate study effect sizes and type I error rate, especially when these studies have underpowered designs. Indeed, the overall negative effect observed in this meta-analysis (Cohen’s *d*_*z*_ = − 0.54) might be driven by some studies reporting inflated large effects and with high standard error due to study small sample sizes (see Fig. 2). Due to the unreliability of the cumulative evidence from experimental studies, there is no certainty of a causal effect, just as there is no certainty of its absence.

### Low Replicability

The power analysis revealed that even considering *d*_*z*_ = − 0.54 as the true effect size, only three studies achieved the considered adequate power of 80%, and only two others would be close (see Fig. 4). The median power of the literature indicates that if we were to conduct 10 exact replications, only ~ 4 out 10 studies would find the expected effect. If we assume the more optimistic adjusted estimate (among all the publication bias methods we applied), these results would be even more dramatic and all studies would be underpowered to detect the adjusted effect. Indeed, a sample size of 63 participants would be required to find a true effect size of − 0.36 given an intended power of 80% and a paired *t*-test. The *z*-curve analysis adds further support to the above results, since the expected discovery rate was .51, 95% CI [.07, .71], which corresponds to the long-run relative frequency of statistically significant results. Therefore, in the future, the sample size should be significantly increased, rather than performing exact replicates. The problem of underpowered studies stems from three main issues.

First, just by the definition of statistical power, if a study has an underpowered design, it has a low probability of detecting a significant effect even if there is one to be found (or the null hypothesis is false) [103]. This is reflected in the observed discovery rate for this literature, which was estimated to be 59%—this is the percentage of articles providing a significant result assuming there is a significant effect to be found. One consideration of this value is that the observed discovery rate does not distinguish between true and false discoveries. Second, studies with low power designs are more likely to produce overestimated effect sizes [29, 104–106]. This will result in literature filled with exaggerated effect estimates if significant original findings are more likely to be published. As far as we know, only one preregistered and replication study to date has been conducted, and the reported effect size was substantially lower and in the opposite direction of the original study [23, 24]. Though replication efforts are still very scarce in the field [107], data from similar disciplines such as psychology show that only half of the original studies were replicated [106, 108] and this would be in agreement with the expected replicability rate of .54 (see Fig. 3). Third, the presence of studies with underpowered designs to find the effect of interest may lead to a bias in the literature due to the increased proportion of false positives [109, 110]. Altogether, the presence of studies with underpowered designs hinders the replicability of scientific results and if only studies with significant results were going to replicate, only 54% of them would yield another significant result. Despite these limitations, the results obtained from studies with underpowered designs have usually been taken at face value. In the past, power issues had been overlooked in the evaluation of results and whenever an effect was significant, it was assumed that the study had enough power [111]. The result is that there has been limited incentive to conduct studies with adequate power [112–114]. Meta-analyses may minimize some of the shortcomings of low-power studies, but they cannot provide a realistic picture of the literature as a whole from a set of low-powered studies [115]. In light of this, the aphorism “Extraordinary claims require extraordinary evidence” may be applicable.

### This Effect Cannot be Discarded

Although it might sound cliché, absence of evidence of an effect does not necessarily prove its absence. In line with our results, another caveat in the literature is effect-size heterogeneity [1–3, 5]. Effect-size heterogeneity refers to the variance in true effect sizes underlying the different studies—that is, there is no single true effect size but rather there is a distribution of true effect sizes. Even when the mean distribution of the true effect size is negative, it is likely that some studies yield effect size estimates around zero or even positive. Heterogeneity is not only reflected on the results of *Q*-statistic test and *I*^2^ estimate but also on the 95% prediction interval as its width accounts for the uncertainty of the summary estimate, the estimate of between study standard deviation in the true effect, and the uncertainty in the between study standard deviation estimate itself. Although the prediction interval is below zero [− 1.32, 0.24] and thus indicating the effect will be detrimental in most settings, the interval overlaps zero and so in some studies the effect may actually be nondetrimental. Then, as we are unaware of the true effect, the results of future implementations are unclear. This finding is masked when we focus only on the average effect and its confidence interval. However, its width will be also enlarged by bias such as publication bias and studies with underpowered designs, in addition to that caused by genuine effect. Therefore, it is possible that performing a mental exertion task does not affect all types of exercise or that the fitness level of participants might mediate its effect. However, the actual presence of these moderators should be interpreted with caution in a set of studies with low statistical power and publication bias.

## Final Remarks

Studies conducted so far have not provided reliable evidence that a causal effect exists, but it is also not certain that one does not exist. It is not only this field of research that suffers from publication bias and low statistical power [37, 101]. In fact, numerous voices have recently highlighted this problem in sport science literature [37, 101, 116–119]. However, this should not be used as an excuse to ignore publication bias and low statistical power. Moreover, as we have seen, meta-analyses are not the ultimate tool to solve the problem of low power. Despite the potential for meta-analyses to mitigate some shortcomings of individual studies, results are largely dependent on the quality of the included reports. Moreover, several sources of publication bias (e.g., small-study effects and selective reporting based on *p*-values) should be considered when performing meta-analyses, as each method is built over specific assumptions [39]. An intervention is sometimes considered to be effective or not based solely on its estimated effect size in a meta-analysis, rather than considering the quality of the primary studies and publication bias. Results from a meta-analysis that shows a high selection bias and low replication rate need to be verified independently in experiments with larger samples (ideally in a multilab study [120]). Nonetheless, the possible negative effects that mental exertion could have on human physical performance cannot be ruled out, but the current evidence suggests that perhaps expectations about this effect should be reduced [1–3, 5].

At a theoretical level, this literature would benefit from integration of other approaches. The literature has assumed that the increased subjective feeling of mental fatigue affects exercise performance and it is mediated by perception of effort. Indeed, mental exertion may increase subjective feelings of mental fatigue, but mental fatigue and perception of effort may be influenced by other cognitive processes [121]. In addition, these studies usually employ standard tasks, and the cognitive load is not adapted to individual abilities [15]. Because they are not adapted, participants can be anywhere between the boredom and distress spectrum [16]. If there is insufficient engagement in the task, boredom can arise and it can modify the behavior of an individual [15, 122]. As a result of boredom, people might perceive the cost/value of subsequent activities differently [14, 123]. When bored, people search for alternative tasks, especially ones that they enjoy. Hence, exercise might be considered as a more rewarding activity and we should not expect a decrease in performance [123–125].

Finally, researchers cannot survive as transparent individuals in a system in which the lack of Open Science practices is the norm [126] and we strongly encourage researchers to preregister study protocols, conduct pre-study power calculations for sample size justification, and make data and materials publicly available to improve credibility [59, 127–129]. Considering the present findings, we encourage caution when making claims or making recommendations on how to counteract the detrimental effects of mental fatigue on exercise performance.

## Footnotes

1 Readers should note that there is much debate about how to define mental fatigue, an issue that goes far beyond the purpose of this article. In this instance, we opted to use a definition we believe describes well what has been studied in the literature about mental fatigue and exercise. It is understandable that any definition is subject to discussion [6–8].

2 Our use of the concept of mental exertion is intended to avoid the confusion between the effect of performing a cognitive task and its possible interpretation.

3 Researchers should ensure that raw data is made publicly available on public repositories, such as the Open Science Framework to facilitate reproducibility and reuse of data. The statement “data will be made available upon request” is outdated and, in most cases, implies that raw data will not be shared [59, 60].

## Declarations

### Data Availability

Ll datasets created are available at https://osf.io/5zbyu/.

### Code Availability

R scripts to analyze data and create figures are available at https://osf.io/5zbyu/.
